# Crystal structure of (*E*)-2-[1-(1,3-benzodioxol-5-yl)ethyl­idene]-*N*-ethyl­hydra­zine-1-carbo­thio­amide

**DOI:** 10.1107/S2056989015003837

**Published:** 2015-02-28

**Authors:** Adriano Bof de Oliveira, Renan Lira de Farias, Christian Näther, Inke Jess

**Affiliations:** a Departamento de Química, Universidade Federal de Sergipe, Av. Marechal Rondon s/n, Campus, 49100-000 São Cristóvão-SE, Brazil; b Institut für Anorganische Chemie, Christian-Albrechts-Universität zu Kiel, Max-Eyth Strasse 2, D-24118 Kiel, Germany

**Keywords:** crystal structure, thio­semicarbazone, benzo[*d*][1,3]dioxole, N—H⋯S hydrogen bonds

## Abstract

In the title compound, C_12_H_15_N_3_O_2_S, the 1,3-benzdioxole fragment is nearly planar [the maximum deviation being 0.0515 (14) Å], the N—N—C(=S)—N fragment is also nearly planar [the maximum deviation being 0.0480 (10) Å], and the dihedral angle between their mean planes is 23.49 (10)°. In the crystal, mol­ecules are linked by pairs of N—H⋯S hydrogen bonds, forming inversion dimers. The dimers are stacked along the *a* axis with neighbouring columns having the same direction; however, the mol­ecules show different orientations leading to a centrosymmetric arrangement. In the crystal, the methyl­ene group of the ethyl substituent and the terminal methyl H atoms are disordered over two sets of sites and were refined using a split model with an occupancy ratio of 0.5:0.5.

## Related literature   

For one of the first reports of the synthesis of thio­semicarbazone derivatives, see: Freund & Schander (1902 ▸). For one of the first reports of 3′,4′-(methyl­enedi­oxy)aceto­phenone extraction from the South American *Aniba rosaeodora* tree, see: Mors *et al.* (1957 ▸). For the crystal structures of two derivatives of the title compound, see: Oliveira *et al.* (2013 ▸, 2015 ▸).
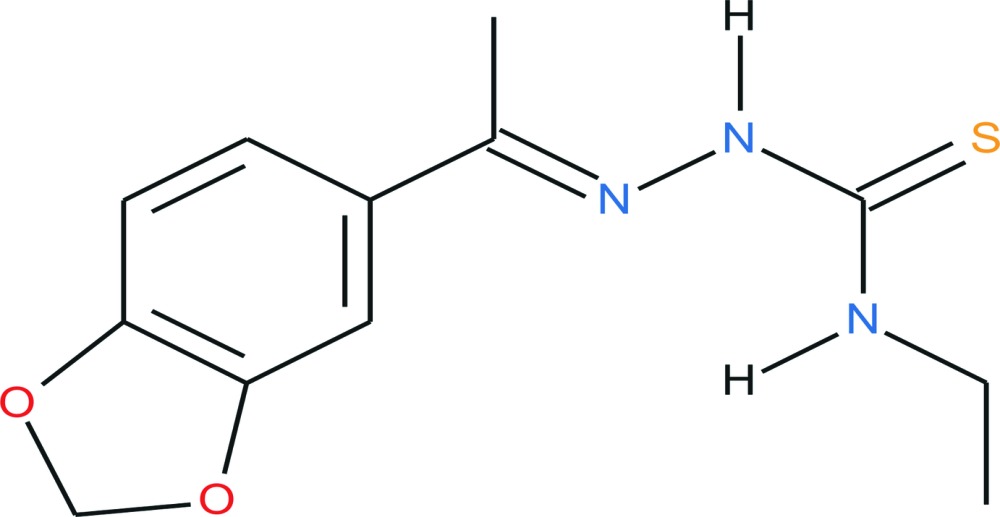


## Experimental   

### Crystal data  

C_12_H_15_N_3_O_2_S
*M* *_r_* = 265.33
Triclinic, 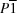

*a* = 5.7207 (3) Å
*b* = 10.6225 (6) Å
*c* = 10.8103 (6) Å
α = 83.908 (5)°
β = 79.913 (5)°
γ = 87.029 (5)°
*V* = 642.74 (6) Å^3^
*Z* = 2
Mo *K*α radiation
μ = 0.25 mm^−1^
*T* = 250 K
0.15 × 0.15 × 0.10 mm

### Data collection  

Stoe IPDS-1 diffractometer
9389 measured reflections
2811 independent reflections
2288 reflections with *I* > 2σ(*I*)
*R*_int_ = 0.042

### Refinement  

*R*[*F* ^2^ > 2σ(*F* ^2^)] = 0.039
*wR*(*F* ^2^) = 0.116
*S* = 1.03
2811 reflections
173 parameters
H-atom parameters constrained
Δρ_max_ = 0.28 e Å^−3^
Δρ_min_ = −0.23 e Å^−3^

### 

Data collection: *X-AREA* (Stoe & Cie, 2008 ▸); cell refinement: *X-AREA*; data reduction: *X-RED32* (Stoe & Cie, 2008 ▸); program(s) used to solve structure: *SHELXS97* (Sheldrick, 2008 ▸); program(s) used to refine structure: *SHELXL2013-2* (Sheldrick, 2015 ▸); molecular graphics: *DIAMOND* (Brandenburg, 2006 ▸); software used to prepare material for publication: *publCIF* (Westrip, 2010 ▸).

## Supplementary Material

Crystal structure: contains datablock(s) I, publication_text. DOI: 10.1107/S2056989015003837/xu5837sup1.cif

Structure factors: contains datablock(s) I. DOI: 10.1107/S2056989015003837/xu5837Isup2.hkl

Supporting information file. DOI: 10.1107/S2056989015003837/xu5837Isup3.cml

**Fig. 1.**
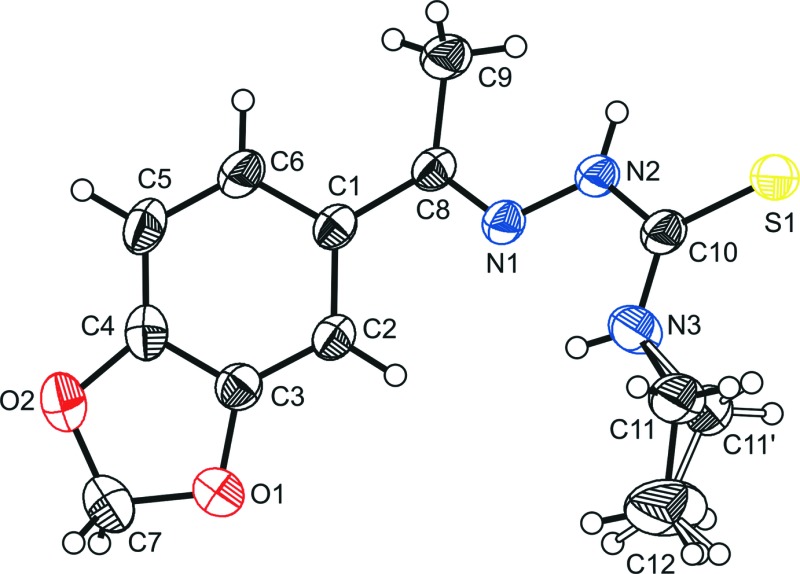
The molecular structure of the title compound with labeling and displacement ellipsoids drawn at the 40% probability level. Disorder is shown with full and open bonds.

. DOI: 10.1107/S2056989015003837/xu5837fig1.tif
The mol­ecular structure of the title compound with labeling and displacement ellipsoids drawn at the 40% probability level. Disorder is shown with full and open bonds.

**Fig. 2.**
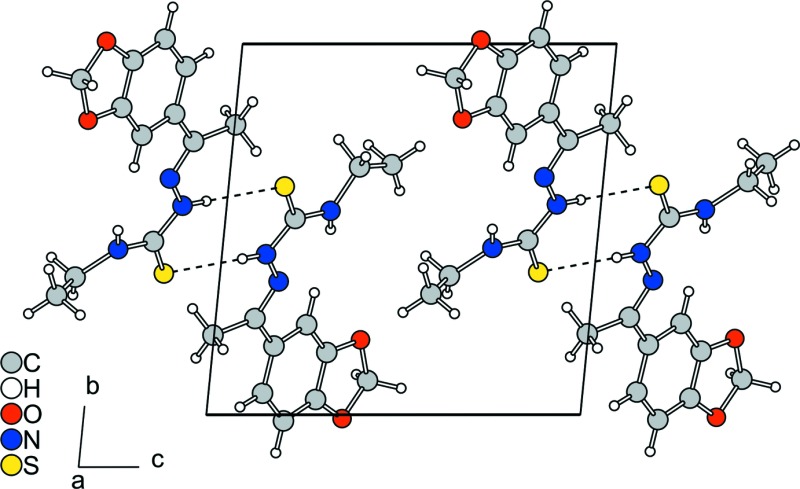
Crystal structure of the title compound with hydrogen bonding shown as dashed lines (see Table 1 for details). Disordered atoms are not shown for clarity.

. DOI: 10.1107/S2056989015003837/xu5837fig2.tif
Crystal structure of the title compound with hydrogen bonding shown as dashed lines (see Table 1 for details). Disordered atoms are not shown for clarity.

CCDC reference: 1051034

Additional supporting information:  crystallographic information; 3D view; checkCIF report

## Figures and Tables

**Table 1:** Hydrogen-bond geometry (, )

*D*H*A*	*D*H	H*A*	*D* *A*	*D*H*A*
N2H1*N*2S1^i^	0.87	2.72	3.5842(14)	175

Symmetry code: (i) 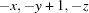.

## supplementary crystallographic information

### S1. Structural commentary

Concerning our inter­est and on going research on thio­semicarbazone derivatives from natural products, we report herein the synthesis and crystal structure of 1-(2*H*-1,3-benzodioxol-5-yl)ethanone 4-ethyl­thio­semicarbazide. The carbonyl­ated precursor is a secondary metabolite from Amazonian Magnoliid trees that belong to the *Lauraceae* family, the *Aniba rosaeodora*, (Mors *et al.*, 1957).

The molecular structure of the title compound, which matches the asymmetric unit, is not planar [the mean deviation from planarity for non-H atoms, and excluding the disordered C11/C11' entity, amounts to 0.3794 (17) Å for C5]. The maximum deviation from the mean plane of the non-H atoms of the 1,3-benzodioxole fragment amounts to 0.0515 (14) Å for C7 and for the N1/N2/C10/S1/N3 fragment amounts 0.0480 (10) Å for N2, with the dihedral angle between the planes being 23.49 (10)°.

In the crystal, the molecules are connected by pairs of N2—*H1N2*···S1 inter­molecular hydrogen bonds building dimers. The dimers are stacked along *a*-axis and although the neighbour columns have the same direction, the dimeric units show different orientations leading to a centrosymmetric structure (Figure 2 and Table 1).

### S1.1. Synthesis and crystallization

Starting materials are commercially available and were used without further purification. The synthesis of the title compound was adapted from a previously procedure (Freund & Schander, 1902). In a hydro­chloric acid catalized reaction, a mixture of 3',4'-(methyl­ene­dioxy)­aceto­phenone (10 mmol) and 4-ethyl-3-thio­semicarbazide (10 mmol) in ethanol (80 mL) was refluxed for 4 h. After cooling and filtering, the title compound was obtained. Colourless crystal grown in DMSO by the slow evaporation of the solvent.

### S1.2. Refinement

The C—H H atoms were positioned with idealized geometry (methyl H atoms were allowed to rotate but not to tip) and refined isotropic with *U*_iso_(H) = 1.2*U*_eq_(C) (1.5 for methyl H atoms) using a riding model with C—H = 0.94 Å for aromatic, C—H = 0.98 Å for methyl­ene and C—H = 0.97 Å for methyl H atoms. The N—H H atoms were located in a difference map and were refined isotropic with varying coordinates in the beginning. Finally, the N—H distances were set to ideal values of 0.87 Å and they were refined isotropic with *U*_iso_(H) = 1.2*U*_eq_(N) using a riding model. The methyl­ene C atom C11 is disordered in two orientations and were refined using a split model with occupancy of 0.5:0.5.

### Crystal data

**Table:**

C_12_H_15_N_3_O_2_S	*Z* = 2
*M**_r_* = 265.33	*F*(000) = 280
Triclinic, *P*1	*D*_x_ = 1.371 Mg m^−^^3^
*a* = 5.7207 (3) Å	Mo *K*α radiation, λ = 0.71073 Å
*b* = 10.6225 (6) Å	Cell parameters from 9389 reflections
*c* = 10.8103 (6) Å	θ = 1.9–27.0°
α = 83.908 (5)°	µ = 0.25 mm^−^^1^
β = 79.913 (5)°	*T* = 250 K
γ = 87.029 (5)°	Parallelepiped, colourless
*V* = 642.74 (6) Å^3^	0.15 × 0.15 × 0.10 mm

### Data collection

**Table:**

Stoe IPDS-1 diffractometer	2288 reflections with *I* > 2σ(*I*)
Radiation source: fine-focus sealed tube, Stoe IPDS-1	*R*_int_ = 0.042
Graphite monochromator	θ_max_ = 27.0°, θ_min_ = 1.9°
φ scans	*h* = −7→7
9389 measured reflections	*k* = −13→13
2811 independent reflections	*l* = −13→13

### Refinement

**Table:**

Refinement on *F*^2^	Primary atom site location: structure-invariant direct methods
Least-squares matrix: full	Secondary atom site location: difference Fourier map
*R*[*F*^2^ > 2σ(*F*^2^)] = 0.039	Hydrogen site location: inferred from neighbouring sites
*wR*(*F*^2^) = 0.116	H-atom parameters constrained
*S* = 1.03	*w* = 1/[σ^2^(*F*_o_^2^) + (0.0649*P*)^2^ + 0.1118*P*] where *P* = (*F*_o_^2^ + 2*F*_c_^2^)/3
2811 reflections	(Δ/σ)_max_ < 0.001
173 parameters	Δρ_max_ = 0.28 e Å^−^^3^
0 restraints	Δρ_min_ = −0.23 e Å^−^^3^

### Special details

**Table:**

Geometry. All e.s.d.'s (except the e.s.d. in the dihedral angle between two l.s. planes) are estimated using the full covariance matrix. The cell e.s.d.'s are taken into account individually in the estimation of e.s.d.'s in distances, angles and torsion angles; correlations between e.s.d.'s in cell parameters are only used when they are defined by crystal symmetry. An approximate (isotropic) treatment of cell e.s.d.'s is used for estimating e.s.d.'s involving l.s. planes.
Refinement. Refinement of *F*^2^ against ALL reflections. The weighted *R*-factor *wR* and goodness of fit *S* are based on *F*^2^, conventional *R*-factors *R* are based on *F*, with *F* set to zero for negative *F*^2^. The threshold expression of *F*^2^ > σ(*F*^2^) is used only for calculating *R*-factors(gt) *etc*. and is not relevant to the choice of reflections for refinement. *R*-factors based on *F*^2^ are statistically about twice as large as those based on *F*, and *R*- factors based on ALL data will be even larger.

### Fractional atomic coordinates and isotropic or equivalent isotropic displacement parameters (Å^2^)

**Table:**

	*x*	*y*	*z*	*U*_iso_*/*U*_eq_	Occ. (<1)
C1	0.7435 (3)	0.18956 (14)	0.16339 (15)	0.0407 (3)	
C2	0.8619 (3)	0.24715 (14)	0.24537 (15)	0.0428 (3)	
H2	0.8352	0.3334	0.2575	0.051*	
C3	1.0167 (3)	0.17309 (16)	0.30646 (16)	0.0457 (4)	
C4	1.0618 (3)	0.04644 (16)	0.28893 (17)	0.0516 (4)	
C5	0.9543 (4)	−0.01187 (16)	0.20857 (19)	0.0581 (5)	
H5	0.9863	−0.0977	0.1962	0.070*	
C6	0.7937 (3)	0.06224 (16)	0.14522 (17)	0.0515 (4)	
H6	0.7174	0.0250	0.0887	0.062*	
O1	1.1441 (2)	0.20752 (13)	0.39317 (13)	0.0621 (4)	
C7	1.2882 (3)	0.0980 (2)	0.4229 (2)	0.0615 (5)	
H7A	1.4562	0.1148	0.3917	0.074*	
H7B	1.2668	0.0771	0.5146	0.074*	
O2	1.2191 (3)	−0.00457 (13)	0.36476 (15)	0.0698 (4)	
C8	0.5558 (3)	0.26302 (14)	0.10543 (15)	0.0407 (3)	
C9	0.4637 (3)	0.21798 (17)	−0.00254 (18)	0.0541 (4)	
H9A	0.3116	0.1794	0.0280	0.081*	
H9B	0.5755	0.1560	−0.0415	0.081*	
H9C	0.4440	0.2892	−0.0643	0.081*	
N1	0.4771 (2)	0.36261 (12)	0.15902 (13)	0.0420 (3)	
N2	0.2949 (2)	0.43540 (12)	0.11811 (13)	0.0432 (3)	
H1N2	0.2234	0.4229	0.0560	0.052*	
C10	0.2019 (3)	0.53176 (15)	0.18658 (16)	0.0461 (4)	
S1	−0.03625 (8)	0.61725 (4)	0.15015 (5)	0.05451 (17)	
N3	0.3122 (3)	0.55269 (18)	0.27906 (18)	0.0732 (5)	
H1N3	0.4362	0.5027	0.2843	0.088*	
C11	0.2171 (16)	0.6290 (8)	0.3858 (8)	0.071 (2)	0.5
H11A	0.1138	0.6987	0.3576	0.085*	0.5
H11B	0.1232	0.5758	0.4544	0.085*	0.5
C11'	0.2731 (18)	0.6716 (7)	0.3447 (8)	0.0675 (19)	0.5
H11C	0.2989	0.7447	0.2812	0.081*	0.5
H11D	0.1073	0.6766	0.3872	0.081*	0.5
C12	0.4155 (6)	0.6790 (3)	0.4304 (3)	0.1149 (12)	
H12A	0.5616	0.6353	0.3959	0.172*	0.5
H12B	0.3914	0.6663	0.5219	0.172*	0.5
H12C	0.4259	0.7689	0.4032	0.172*	0.5
H12D	0.4978	0.5980	0.4444	0.172*	0.5
H12E	0.3205	0.7007	0.5092	0.172*	0.5
H12F	0.5307	0.7438	0.3993	0.172*	0.5

### Atomic displacement parameters (Å^2^)

**Table:**

	*U*^11^	*U*^22^	*U*^33^	*U*^12^	*U*^13^	*U*^23^
C1	0.0423 (7)	0.0354 (7)	0.0438 (8)	0.0004 (6)	−0.0029 (6)	−0.0089 (6)
C2	0.0447 (8)	0.0345 (7)	0.0491 (8)	−0.0006 (6)	−0.0058 (6)	−0.0077 (6)
C3	0.0450 (8)	0.0449 (8)	0.0467 (8)	−0.0031 (6)	−0.0063 (6)	−0.0043 (6)
C4	0.0492 (9)	0.0433 (9)	0.0588 (10)	0.0060 (7)	−0.0064 (7)	0.0028 (7)
C5	0.0666 (11)	0.0355 (8)	0.0716 (12)	0.0095 (7)	−0.0103 (9)	−0.0106 (8)
C6	0.0577 (10)	0.0395 (8)	0.0594 (10)	0.0034 (7)	−0.0105 (8)	−0.0161 (7)
O1	0.0644 (8)	0.0599 (8)	0.0684 (8)	0.0018 (6)	−0.0293 (7)	−0.0074 (6)
C7	0.0524 (10)	0.0694 (12)	0.0607 (11)	0.0010 (9)	−0.0142 (8)	0.0083 (9)
O2	0.0699 (9)	0.0565 (8)	0.0853 (10)	0.0114 (6)	−0.0292 (7)	0.0024 (7)
C8	0.0417 (7)	0.0357 (7)	0.0449 (8)	−0.0012 (6)	−0.0050 (6)	−0.0095 (6)
C9	0.0538 (9)	0.0522 (10)	0.0617 (10)	0.0070 (7)	−0.0156 (8)	−0.0245 (8)
N1	0.0417 (6)	0.0370 (6)	0.0485 (7)	0.0033 (5)	−0.0086 (5)	−0.0108 (5)
N2	0.0430 (7)	0.0398 (7)	0.0501 (7)	0.0048 (5)	−0.0128 (5)	−0.0150 (5)
C10	0.0464 (8)	0.0403 (8)	0.0539 (9)	0.0033 (6)	−0.0106 (7)	−0.0150 (7)
S1	0.0481 (2)	0.0521 (3)	0.0700 (3)	0.01332 (18)	−0.02210 (19)	−0.0241 (2)
N3	0.0788 (11)	0.0749 (11)	0.0815 (11)	0.0404 (9)	−0.0444 (9)	−0.0480 (9)
C11	0.070 (4)	0.073 (5)	0.081 (6)	0.027 (4)	−0.029 (4)	−0.046 (4)
C11'	0.087 (5)	0.057 (4)	0.068 (5)	0.027 (3)	−0.032 (4)	−0.033 (3)
C12	0.109 (2)	0.133 (3)	0.122 (2)	0.029 (2)	−0.0365 (19)	−0.089 (2)

### Geometric parameters (Å, º)

**Table:**

C1—C6	1.395 (2)	N1—N2	1.3730 (18)
C1—C2	1.410 (2)	N2—C10	1.358 (2)
C1—C8	1.483 (2)	N2—H1N2	0.8699
C2—C3	1.364 (2)	C10—N3	1.315 (2)
C2—H2	0.9400	C10—S1	1.6792 (17)
C3—O1	1.372 (2)	N3—C11	1.489 (9)
C3—C4	1.383 (2)	N3—C11'	1.501 (9)
C4—C5	1.363 (3)	N3—H1N3	0.8700
C4—O2	1.375 (2)	C11—C12	1.453 (10)
C5—C6	1.401 (3)	C11—H11A	0.9800
C5—H5	0.9400	C11—H11B	0.9800
C6—H6	0.9400	C11'—C12	1.347 (10)
O1—C7	1.432 (2)	C11'—H11C	0.9800
C7—O2	1.420 (3)	C11'—H11D	0.9800
C7—H7A	0.9800	C12—H12A	0.9700
C7—H7B	0.9800	C12—H12B	0.9700
C8—N1	1.2832 (19)	C12—H12C	0.9700
C8—C9	1.493 (2)	C12—H12D	0.9700
C9—H9A	0.9700	C12—H12E	0.9700
C9—H9B	0.9700	C12—H12F	0.9700
C9—H9C	0.9700		
			
C6—C1—C2	119.34 (15)	C10—N2—N1	117.50 (13)
C6—C1—C8	121.30 (15)	C10—N2—H1N2	115.4
C2—C1—C8	119.22 (13)	N1—N2—H1N2	126.9
C3—C2—C1	117.54 (14)	N3—C10—N2	115.66 (15)
C3—C2—H2	121.2	N3—C10—S1	123.92 (12)
C1—C2—H2	121.2	N2—C10—S1	120.41 (13)
C2—C3—O1	127.54 (15)	C10—N3—C11	126.2 (4)
C2—C3—C4	122.40 (16)	C10—N3—C11'	123.1 (4)
O1—C3—C4	110.05 (15)	C10—N3—H1N3	113.3
C5—C4—O2	128.69 (16)	C11—N3—H1N3	119.0
C5—C4—C3	121.73 (16)	C11'—N3—H1N3	121.5
O2—C4—C3	109.57 (17)	C12—C11—N3	108.7 (6)
C4—C5—C6	116.86 (15)	C12—C11—H11A	110.0
C4—C5—H5	121.6	N3—C11—H11A	110.0
C6—C5—H5	121.6	C12—C11—H11B	110.0
C1—C6—C5	122.10 (17)	N3—C11—H11B	110.0
C1—C6—H6	118.9	H11A—C11—H11B	108.3
C5—C6—H6	118.9	C12—C11'—N3	114.0 (6)
C3—O1—C7	105.38 (14)	C12—C11'—H11C	108.7
O2—C7—O1	108.49 (15)	N3—C11'—H11C	108.7
O2—C7—H7A	110.0	C12—C11'—H11D	108.7
O1—C7—H7A	110.0	N3—C11'—H11D	108.7
O2—C7—H7B	110.0	H11C—C11'—H11D	107.6
O1—C7—H7B	110.0	C11—C12—H12A	109.5
H7A—C7—H7B	108.4	C11—C12—H12B	109.5
C4—O2—C7	105.90 (14)	H12A—C12—H12B	109.5
N1—C8—C1	114.42 (14)	C11—C12—H12C	109.5
N1—C8—C9	124.48 (15)	H12A—C12—H12C	109.5
C1—C8—C9	121.04 (13)	H12B—C12—H12C	109.5
C8—C9—H9A	109.5	C11'—C12—H12D	109.5
C8—C9—H9B	109.5	C11'—C12—H12E	109.5
H9A—C9—H9B	109.5	H12D—C12—H12E	109.5
C8—C9—H9C	109.5	C11'—C12—H12F	109.5
H9A—C9—H9C	109.5	H12D—C12—H12F	109.5
H9B—C9—H9C	109.5	H12E—C12—H12F	109.5
C8—N1—N2	120.07 (14)		
			
C6—C1—C2—C3	−1.9 (2)	C6—C1—C8—N1	159.25 (15)
C8—C1—C2—C3	173.75 (14)	C2—C1—C8—N1	−16.3 (2)
C1—C2—C3—O1	−177.54 (15)	C6—C1—C8—C9	−18.1 (2)
C1—C2—C3—C4	1.0 (2)	C2—C1—C8—C9	166.30 (15)
C2—C3—C4—C5	0.3 (3)	C1—C8—N1—N2	−176.84 (12)
O1—C3—C4—C5	179.06 (16)	C9—C8—N1—N2	0.4 (2)
C2—C3—C4—O2	−178.38 (15)	C8—N1—N2—C10	173.15 (14)
O1—C3—C4—O2	0.34 (19)	N1—N2—C10—N3	6.2 (2)
O2—C4—C5—C6	177.85 (17)	N1—N2—C10—S1	−174.75 (11)
C3—C4—C5—C6	−0.6 (3)	N2—C10—N3—C11	−164.2 (4)
C2—C1—C6—C5	1.7 (3)	S1—C10—N3—C11	16.8 (5)
C8—C1—C6—C5	−173.86 (16)	N2—C10—N3—C11'	165.3 (4)
C4—C5—C6—C1	−0.4 (3)	S1—C10—N3—C11'	−13.8 (5)
C2—C3—O1—C7	−176.90 (16)	C10—N3—C11—C12	−153.3 (4)
C4—C3—O1—C7	4.46 (18)	C11'—N3—C11—C12	−61.3 (15)
C3—O1—C7—O2	−7.55 (19)	C10—N3—C11'—C12	−175.3 (4)
C5—C4—O2—C7	176.35 (19)	C11—N3—C11'—C12	79.0 (15)
C3—C4—O2—C7	−5.04 (19)	N3—C11'—C12—C11	−73.0 (13)
O1—C7—O2—C4	7.8 (2)	N3—C11—C12—C11'	68.3 (14)

### Hydrogen-bond geometry (Å, º)

**Table:**

*D*—H···*A*	*D*—H	H···*A*	*D*···*A*	*D*—H···*A*
N2—H1*N*2···S1^i^	0.87	2.72	3.5842 (14)	175

Symmetry code: (i) −*x*, −*y*+1, −*z*.
