# Supplementary figures and images for: Crystal structure of (E)-2-[1-(1,3-benzodioxol-5-yl)ethyl­idene]-N-ethyl­hydra­zine-1-carbo­thio­amide
Source: Acta Crystallogr E Crystallogr Commun. 2015 Feb 28;71(Pt 3):o208–9. doi: 10.1107/S2056989015003837 (PMC4350709; doi:10.1107/S2056989015003837)

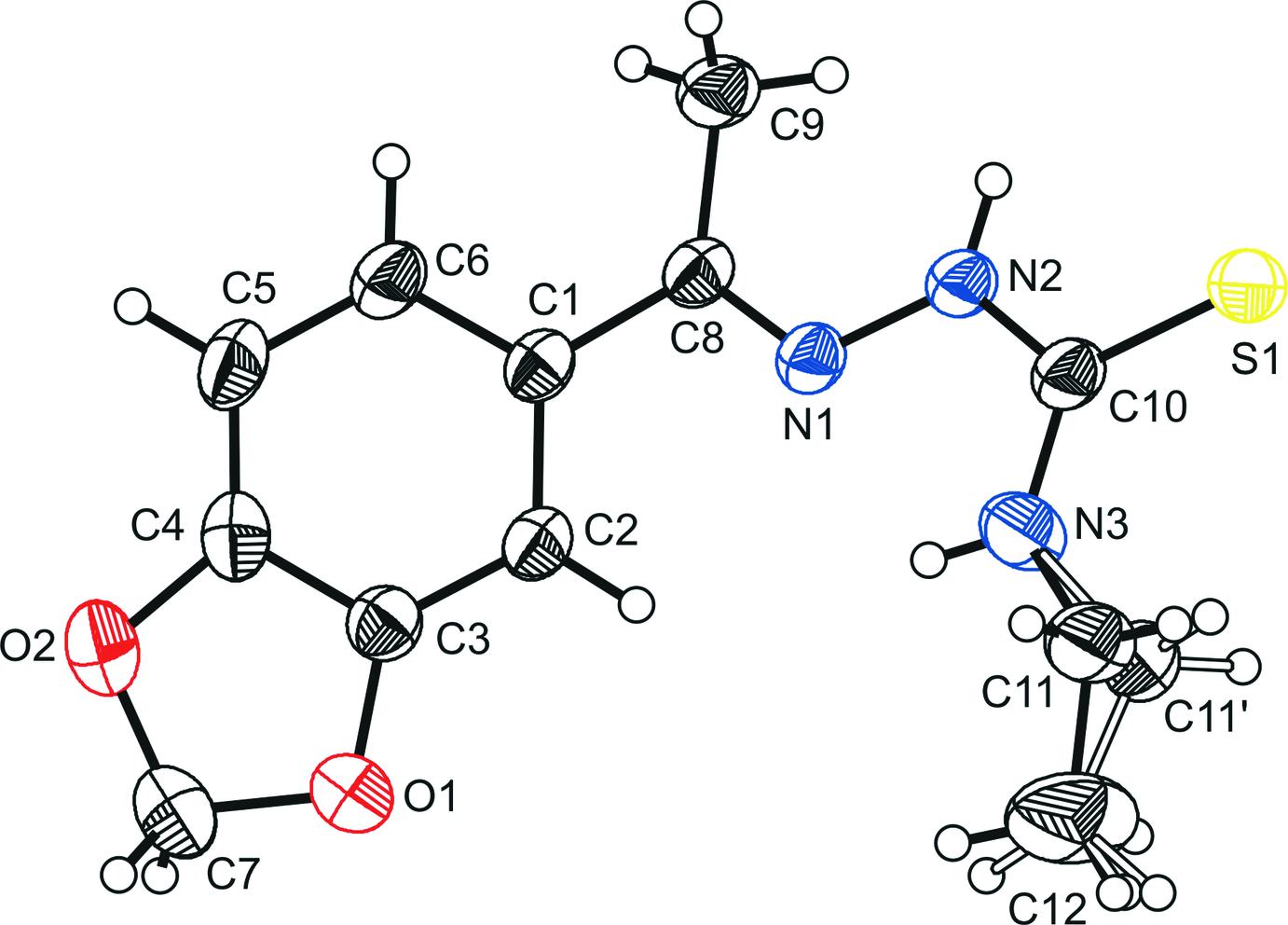

Supplement: Supplementary file 4 [file e-71-0o208-fig1.tif]

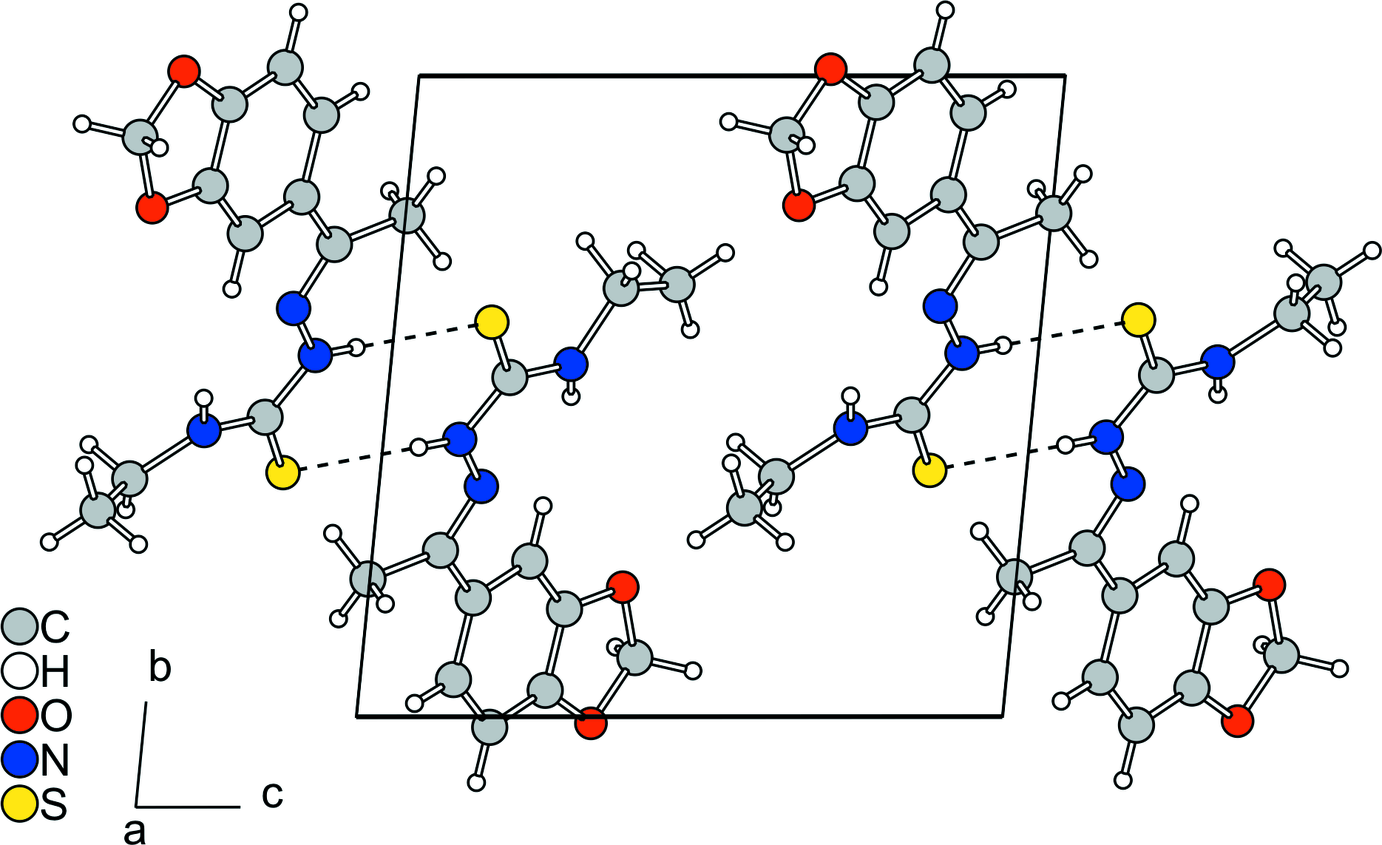

Supplement: Supplementary file 5 [file e-71-0o208-fig2.tif]
